# Endoscopic ultrasound suggestive of a gastric duplication cyst presenting as a gastric subepithelial tumor-like lesion

**DOI:** 10.1055/a-2957-9789

**Published:** 2026-09-28

**Authors:** Koichi Soga, Satsuki Otani, Ou Takagi, Takaya Kobayashi, Ikuhiro Kobori, Kazuyuki Saito, Katsutoshi Sugimoto

**Affiliations:** 1 Department of Gastroenterology 26263 Dokkyo Medical University Saitama Medical CenterKoshigayaSaitamaJapan; 2 Department of Surgery 26263 Dokkyo Medical University Saitama Medical CenterKoshigayaJapan

A 35-year-old woman was referred to our hospital for the evaluation of an incidentally detected protruding gastric lesion on screening upper gastrointestinal endoscopy. She was asymptomatic with no relevant medical history.

Contrast-enhanced computed tomography and T2-weighted magnetic resonance imaging revealed a well-demarcated cystic lesion adjacent to the posterior wall of the upper gastric body, with mild peripheral wall enhancement and heterogeneous intracystic content. No definite mural nodules or solid components were identified ( Figs. 1 and 2 ).

**Fig. 1:**
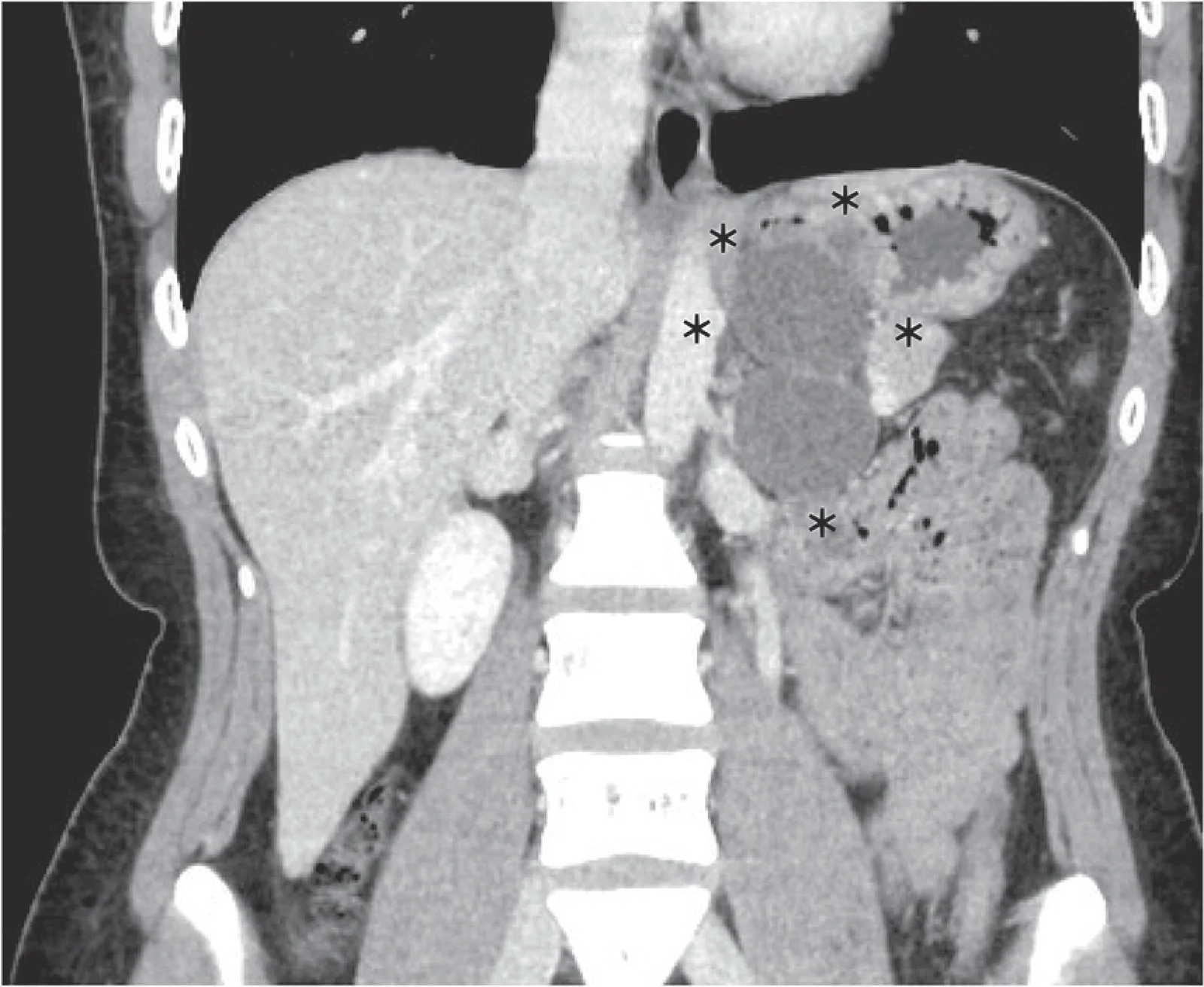
Contrast-enhanced computed tomography findings. Coronal contrast-enhanced computed tomography showing a well-demarcated cystic lesion (asterisks) adjacent to the posterior wall of the upper gastric body. The lesion contains a slightly hyperattenuating intracystic material rather than a simple fluid, and mild enhancement is observed along the cyst wall and peripheral margins. No definite mural nodules, irregular wall thickening, or solid components are observed.

**Fig. 2:**
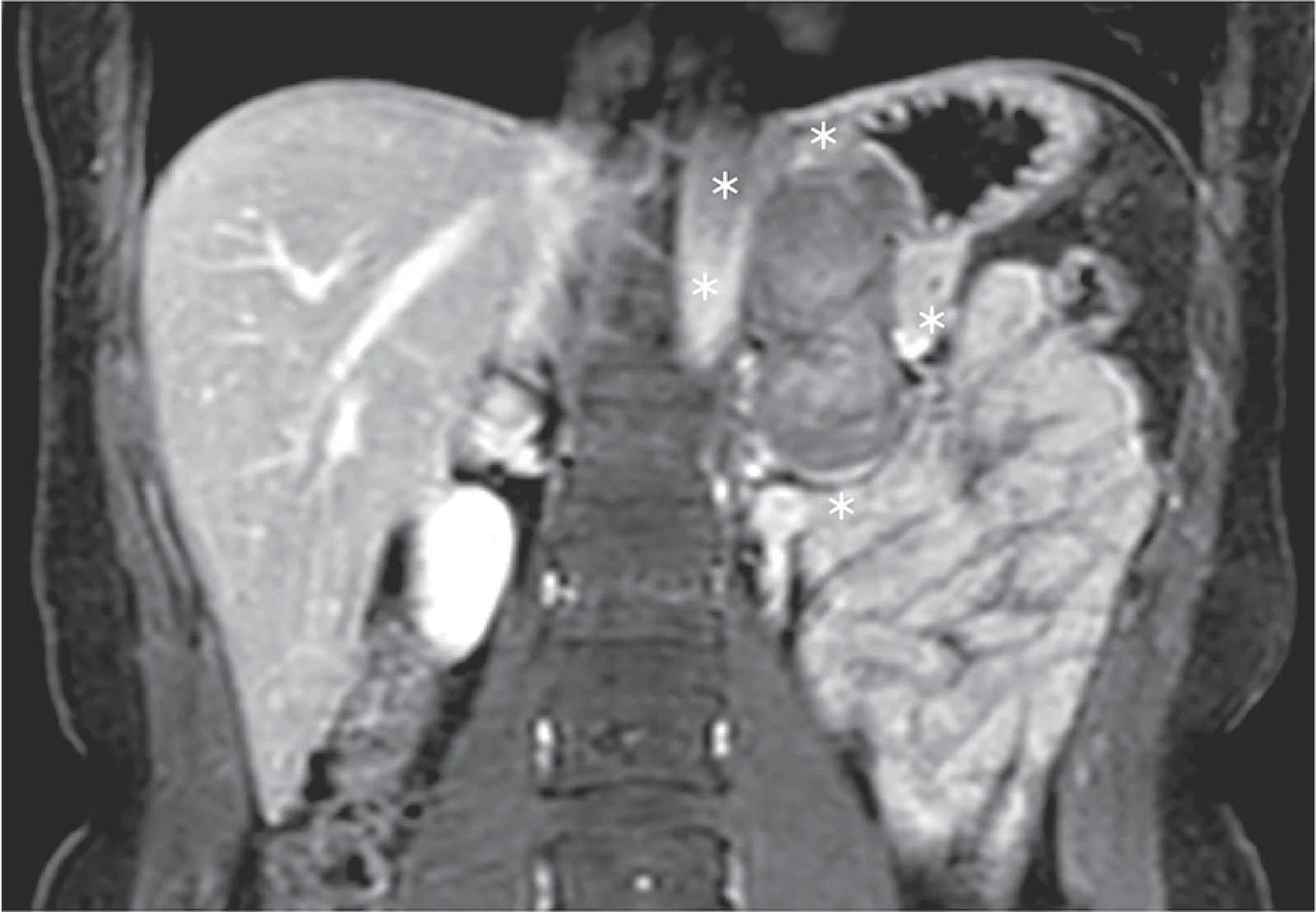
Abdominal magnetic resonance imaging findings. Coronal T2-weighted magnetic resonance imaging showing a well-demarcated cystic lesion (asterisks) adjacent to the posterior wall of the upper gastric body. The lesion does not show homogeneous simple fluid intensity but demonstrates slightly heterogeneous internal signal intensity, suggesting a cystic nature. A low-signal peripheral rim corresponding to the cyst wall was observed. No definite mural nodules or solid components are observed.

Conventional endoscopy revealed a broad, mildly elevated subepithelial tumor-like lesion on the posterior wall of the upper gastric body. The smooth overlying mucosa, without erosion or ulceration, suggested a differential diagnosis of a true gastric subepithelial lesion versus extrinsic compression. Radial endoscopic ultrasound (EUS) was performed using deaerated water instillation into the stomach. The gastric wall five-layer structure was preserved, and the adjacent cystic lesion showed a gut wall-like layered architecture with an inner hyperechoic layer and an outer hypoechoic layer resembling the muscularis propria ( Fig. 3 ). This appearance suggested a double wall sign, a characteristic sonographic finding of gastrointestinal duplication cysts. 1​ ​2​

**Fig. 3:**
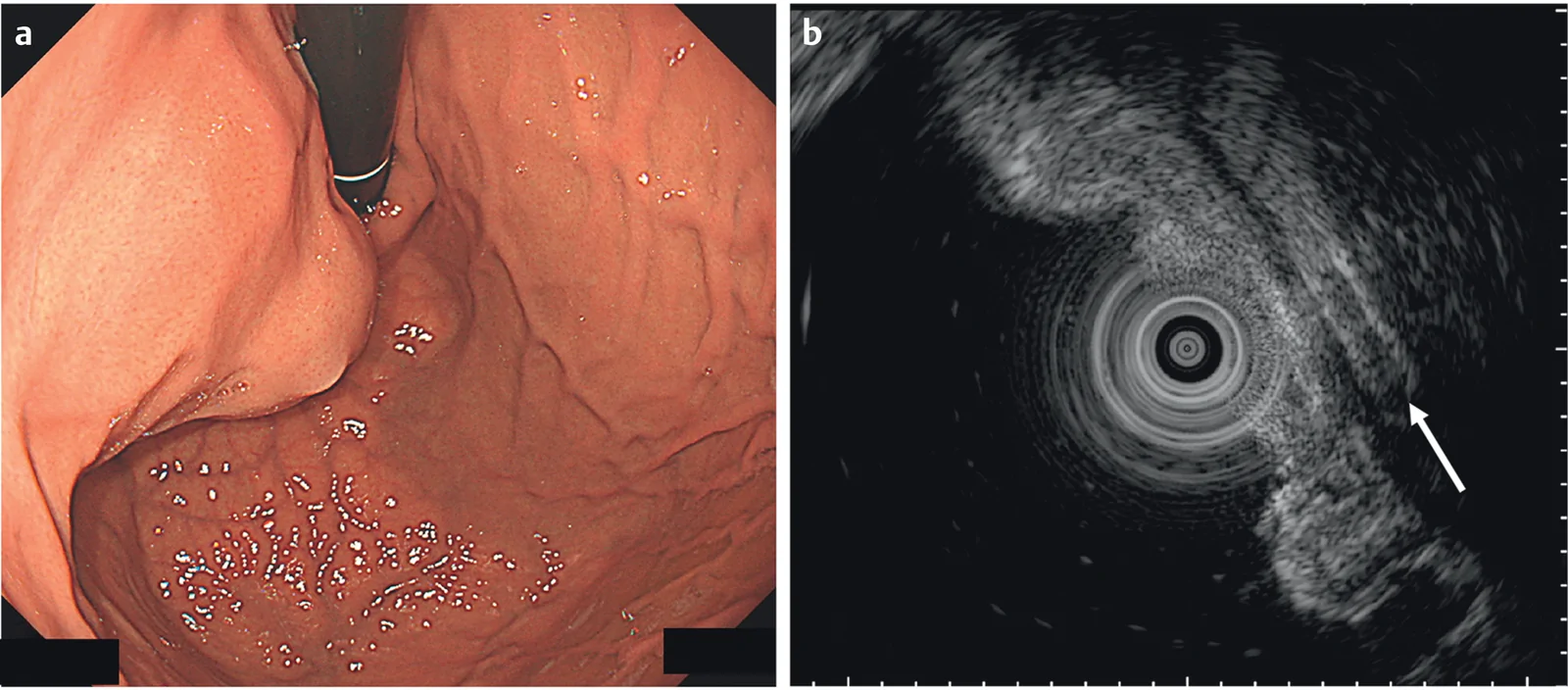
Radial endoscopic ultrasound (EUS) evaluation of a gastric subepithelial tumor-like lesion. ( **a** ) Conventional endoscopy showing a broad, mildly elevated subepithelial tumor-like lesion on the posterior wall of the upper gastric body. The smooth overlying mucosa, without erosion, ulceration, or irregularities. ( **b** ) Radial EUS using a 20-MHz probe was performed using deaerated water instillation into the stomach. The gastric wall five-layer structure is preserved. The lesion is seen as a cystic structure adjacent to the outer aspect of the gastric wall. The cyst wall shows a gut wall-like layered architecture. These findings suggested a double wall sign (arrow), which is the characteristic of a gastrointestinal duplication cyst.

Further evaluation using a convex EUS revealed a cystic lesion attached to the outer aspect of the gastric wall ( Fig. 4 ). The lesion contained fluid with fine internal echoes, and endoscopic manipulation induced swirling of the intracystic contents, confirming its cystic nature. At the interface between the gastric wall and the cystic lesion, the hypoechoic layers appeared to converge focally, suggesting a possible Y-configuration sign ( Fig. 5 ), possibly reflecting splitting or sharing of the muscularis propria 1​ ( Video 1 ).

**Fig. 4:**
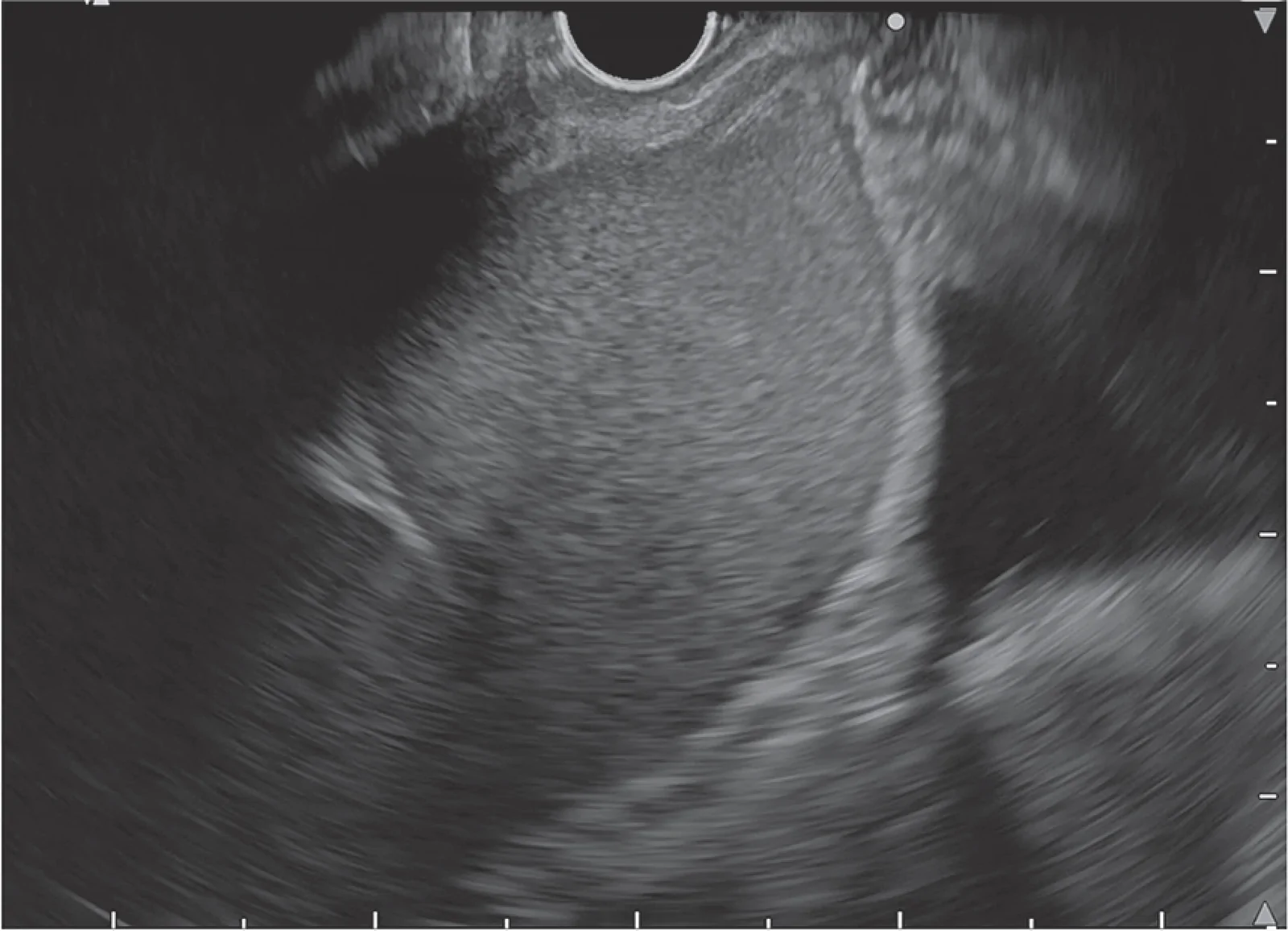
Evaluation of the cystic lesion using convex EUS. Convex EUS demonstrates a cystic lesion adjacent to the outer aspect of the gastric wall, corresponding to a subepithelial tumor-like elevation in the upper gastric body. The lesion appears attached to the gastric wall and contains a fluid, with fine internal echoes. Endoscopic manipulation induces swirling of intracystic contents, supporting the presence of a fluid rather than a solid tumor. The cyst wall shows a gut wall-like layered structure, suggesting a gastrointestinal duplication cyst. No definite mural nodules or solid components are observed.

**Fig. 5:**
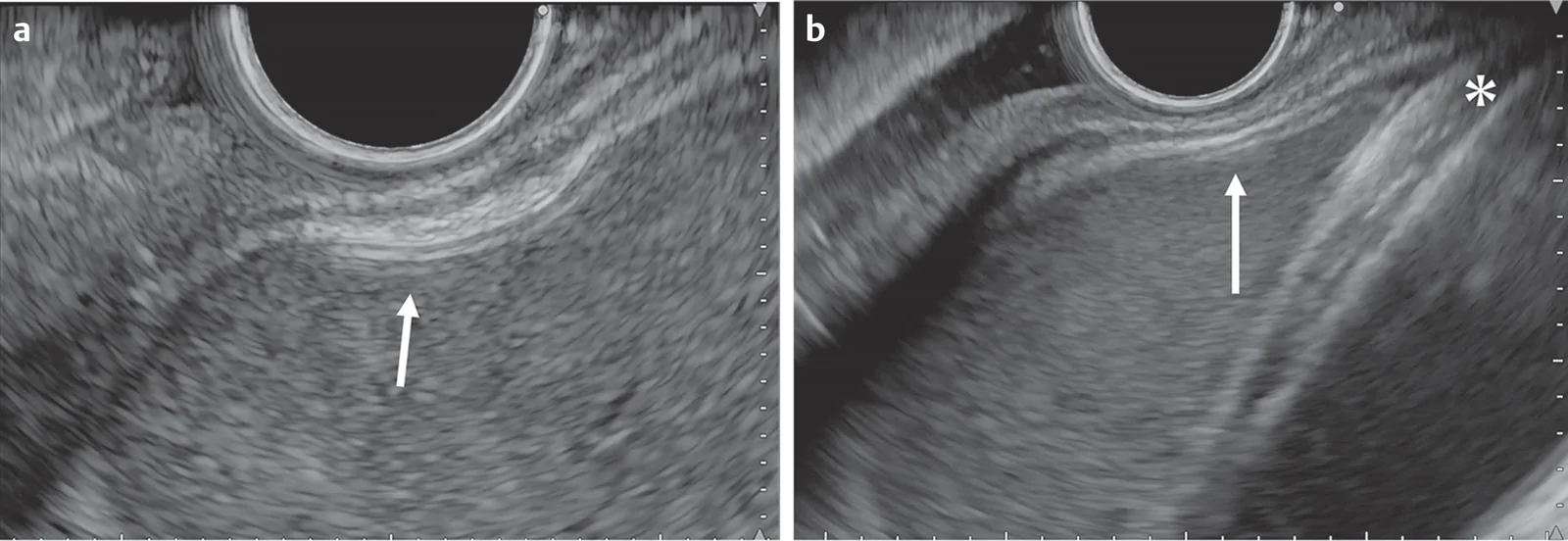
Detailed convex EUS assessment of a cyst wall architecture and its interface with the gastric wall. A subepithelial tumor-like lesion on the posterior wall of the upper gastric body was evaluated using convex EUS. ( **a** ) The gastric wall five-layer structure is preserved. The wall of the extrinsic mass adjacent to the gastric wall shows a gut wall-like layered architecture (arrow). ( **b** ) The interface between the gastric wall and the lesion shows close apposition of the cystic lesion to the gastric wall. The cyst wall demonstrates a double wall sign (arrow), consisting of an inner hyperechoic layer and an outer hypoechoic layer. Furthermore, the hypoechoic layers of the gastric and cyst walls appear to converge focally, suggesting a Y-configuration sign (asterisk). Continuity between the muscularis propria of the gastric wall and the muscular layer of the cyst wall is demonstrated on EUS.

**Video 1** Endoscopic ultrasound findings suggestive of a gastric duplication cyst presenting as a gastric subepithelial tumor-like lesion. A 35-year-old woman was referred for the evaluation of an incidentally detected protruding gastric lesion. Radial and convex EUS with deaerated water instillation demonstrate a cystic lesion adjacent to the posterior wall of the upper gastric body. The lesion shows the gut wall–like layered architecture, a double wall sign, and an internal fluid with fine echoes. Endoscopic manipulation induces swirling of intracystic contents, supporting the cystic nature of the lesion. A possible Y-configuration sign is also observed at the interface between the lesion and gastric wall.


Surgical resection was performed, and histopathological examination confirmed a gastrointestinal duplication cyst. This case highlights that EUS can facilitate preoperative diagnosis by demonstrating cystic contents, gut wall-like architectures, and characteristic signs 2​ 3​ ​4​ .

## Declaration of GenAI Use

The authors confirm that no generative artificial intelligence was used in the preparation of this manuscript.

## Institutional review board approval

Not required for this case report in accordance with institutional policy.

Endoscopy_UCTN_Code_TTT_1AS_2AB
